# Case report: Unruptured sinus of Valsalva aneurysms causing postural angina pectoris and false “pulmonary hypertension”

**DOI:** 10.3389/fcvm.2023.1120633

**Published:** 2023-03-22

**Authors:** Chi Zhou, Jingwen Tao, Sheng Li

**Affiliations:** Division of Cardiology, Department of Internal Medicine, Tongji Hospital, Tongji Medical College of Huazhong University of Science and Technology, Wuhan, China

**Keywords:** sinus of Valsalva aneurysm, postural angina pectoris, pulmonary hypertension, case report, FLNA gene mutation

## Abstract

A 37-year-old woman presented with worsening intermittent chest pain and dyspnea in the previous year. Although the dyspnea was exertion-dependent, her chest pain was heavily dependent on her postural position, worsening in the supine position but alleviated by lying prone or by sitting up and leaning forward. She was cyanotic, and a diastolic murmur in the left third intercostal space was auscultated. An electrocardiogram recorded when she laid flat and had angina pectoris attacks showed ST-segment elevation in the aVR and depression in the II, III, aVF, and V3–V6 leads. However, when she sat up for a few minutes, her symptoms and ST-segment abnormalities disappeared. Echocardiography and cardiac computed tomography angiography revealed large unruptured aneurysms of the left and non-coronary sinuses, along with a dilated aortic root, severe aortic regurgitation, and right ventricular high pressure. Coronary angiography showed ∼90% pulsating stenosis of the left main coronary artery and ∼80% pulsating stenosis of the proximal left circumflex artery, presumably caused by pulsation of the dilated sinus of Valsalva aneurysm under blood pressure. Genetic testing revealed c.1781 C > G nonsense mutations in the *FLNA* gene. The patient underwent surgery, which confirmed dual unruptured left/non-coronary sinus of Valsalva aneurysms. Our case illustrates an unusual postural form of angina pectoris and false “pulmonary hypertension” caused by large dual unruptured left/non-coronary sinus of Valsalva aneurysms.

## Introduction

Sinus of Valsalva aneurysm (SVA) is a rare cardiac abnormality that was first described by Hope in 1839. Its estimated prevalence is 0.09% in the general population (1). It is worth noting that SVA occurs five times more often in Asian than Western populations, with a predominance in men (2). The origin of SVA can be congenital or acquired, with the former being more common. Congenital SVA is caused by defective continuity between the aortic media and aortic valve annulus fibrosis. Previous studies have indicated that mutations in *FBN1* or *MFAP5* may be related to congenital SVA (3). Acquired SVA usually arises from chest trauma, infective endocarditis, syphilis, tuberculosis, aortitis, atherosclerosis, or connective tissue disorders (4). The clinical presentation of SVA varies. Ruptured SVA often causes substernal chest pain, dyspnea, and sudden cardiac arrest, whereas most unruptured SVA cases are asymptomatic (1). Once an unruptured SVA is identified, surgical repair is vital to prevent its rupture, which can lead to myocardial infarction, malignant arrhythmia, heart failure, and pericardial tamponade. The mean survival period for patients with untreated, ruptured SVA is 3.9 years (5). This report describes a case of dual unruptured left/non-coronary SVAs that led to postural angina pectoris and false “pulmonary hypertension”.

## Case presentation

**Figure 1:**
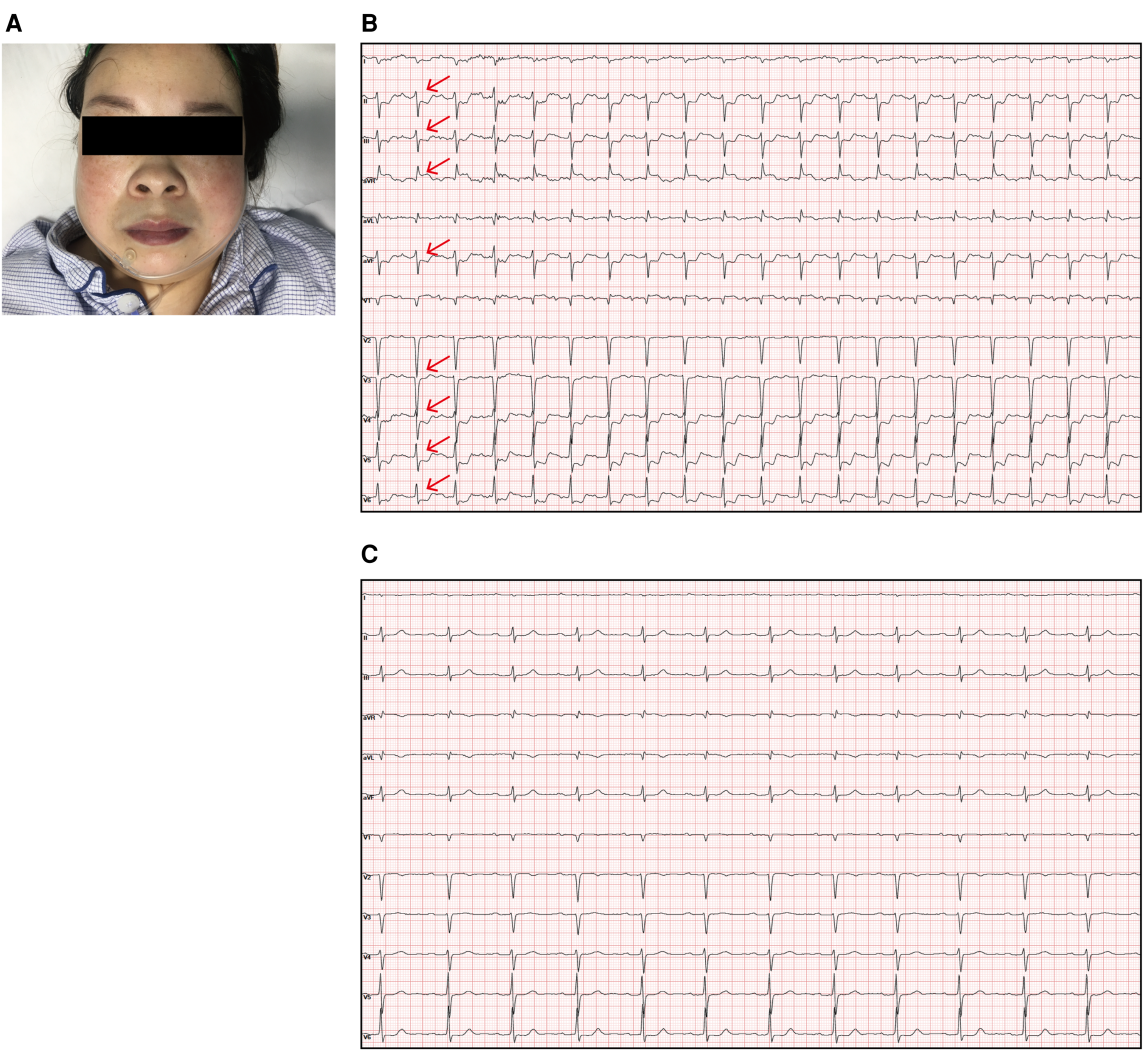
(**A**) The patient was cyanotic. (**B**) Electrocardiogram showing sinus rhythm and obvious ST-segment elevation in the aVR (red arrow) as well as depressions in the II, III, aVF, and V3–V6 leads (red arrow) when she laid flat and experienced angina pectoris. (**C**) The angina pectoris and ST segment abnormalities were relieved when she sat up.

A 37-year-old woman presented with worsening intermittent chest pain and dyspnea for 1 year and was admitted to our hospital. Interestingly, although the dyspnea was exertion-dependent, her chest pain heavily depended on the postural position as it worsened in the supine position but was alleviated by lying prone or sitting up and leaning forward. The patient had moderate anemia due to adenomyosis. She had no history of chest trauma, infective endocarditis, syphilis, tuberculosis, hypertension, diabetes, dyslipidemia, aortic aneurysm, or connective tissue disease. Her vital signs were as follows: body temperature 36.5 °C, blood pressure 130/70 mmHg, pulse 75 beats/min, respiratory rate 20 breaths/min, and oxygen saturation 96% (indoor air). A physical examination revealed lip cyanosis (Figure 1A) and a diastolic murmur at the left third intercostal space was present. Respiratory and abdominal examinations revealed no abnormal findings. Written informed consent for publication was obtained from the patient.

**Figure 2:**
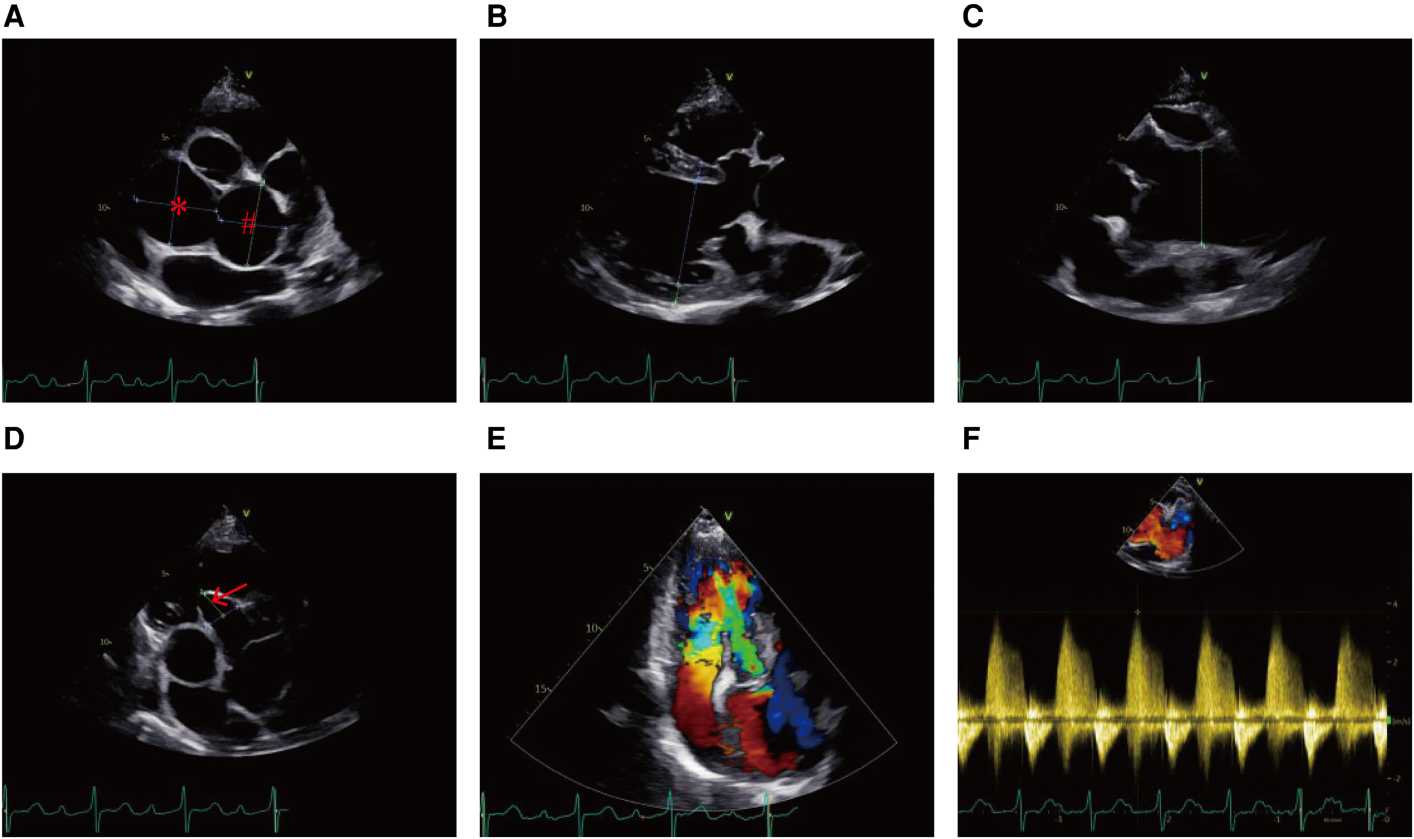
Echocardiogram showing (**A**) the unruptured left SVA (#) and non-coronary SVA (*), (**B**) enlarged left ventricle, (**C**) dilated aortic root, (**D**) ventricular septal membranous aneurysm (red arrow), (**E**) severe aortic regurgitation, and (**F**) right ventricular high pressure.

**Figure 3:**
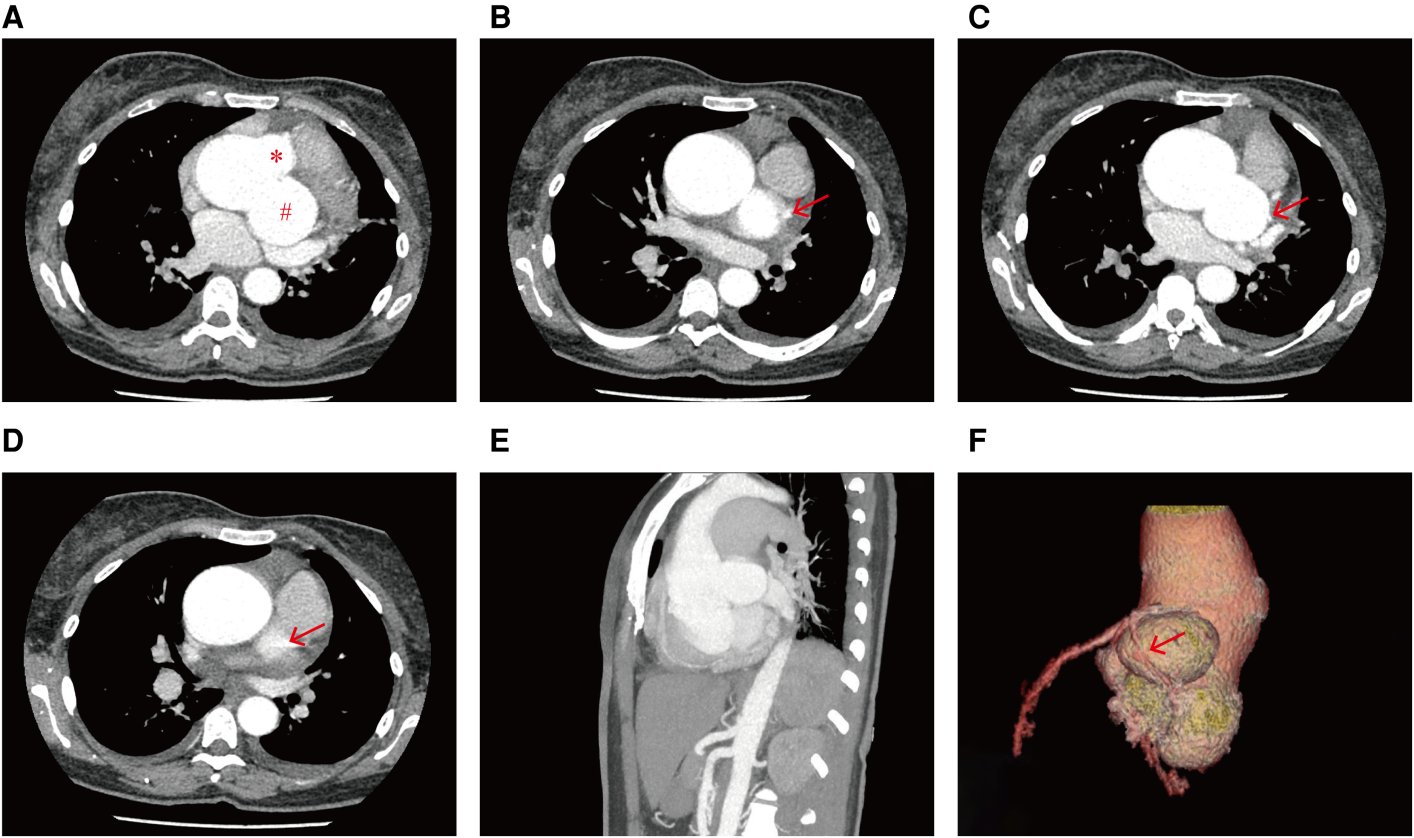
CCTA images showing (**A**) the unruptured left SVA (#) and non-coronary SVA (*), the effect of the dilated left SVA on the (**B**) LM (red arrow) and the (**C**) LCX (red arrow), and (**D**) the dilated left SVA upwardly squeezed the pulmonary trunk (red arrow). (**E**) Sagittal plane view of the dual unruptured left/non-coronary SVAs. (**F**) Three-dimensional reconstruction image showing the dual unruptured left/non-coronary SVAs, passage of the LM and LCX, and compression of the LCX (red arrow).

A hematological evaluation showed a hemoglobin level of 73 g/L (microcytic hypochromic anemia), a white blood cell count of 4.59 × 10^9^/L, and a platelet count of 143 × 10^9^/L. Other laboratory data, including myocardial enzyme levels, NT pro-brain natriuretic peptide levels, coagulation function, and hepatic and renal function were normal. Electrocardiography showed sinus rhythm, obvious ST-segment elevation in the aVR, and depression in the II, III, aVF, and V3–V6 leads when she lay flat and had angina pectoris attacks (Figure 1B). However, when she sat up for a few minutes, her symptoms and ST segment abnormalities disappeared (Figure 1C). Echocardiography revealed large unruptured aneurysms in both the left (47 mm × 43 mm) and non-coronary (44 mm × 35 mm) sinuses (Figure 2A), along with an enlarged left ventricle (58 mm, Figure 2B), a dilated aortic root (52 mm, Figure 2C), a ventricular septal membranous aneurysm (19 mm × 16 mm, Figure 2D), severe aortic regurgitation (Figure 2E), right ventricular high pressure (pressure gradient 54 mmHg, Figure 2F), and decreased left ventricular systolic function (ejection fraction 51%). Coronary angiography showed ∼90% pulsating stenosis of the left main coronary artery (LM) and ∼80% pulsating stenosis of the proximal left circumflex artery (LCX), presumably caused by pulsation of the dilated left SVA under blood pressure (Supplementary Video S1). The aortic root was severely distorted when we attempted to intubate the coronary arteries. Non-obstructive atherosclerosis was observed in the right coronary artery (RCA). Cardiac computed tomography angiography (CCTA) clearly showed a large unruptured left SVA (Figure 3A), which was anatomically close to the LM (Figure 3B) and proximal LCX (Figure 3C), causing severe narrowing of the vessel lumens. The dilated left SVA upwardly squeezed the pulmonary trunk (Figure 3D) and accelerated the maximum pulmonary blood flow to 3.7 m/s. The dilated non-coronary SVA adjoined the right atrium without a crevasse (Figure 3A). The right coronary sinus was normal, and the RCA blood flow was smooth. The sagittal plane view showed dual unruptured left/non-coronary SVAs (Figure 3E). Three-dimensional reconstruction images showed the LM and LCX running along the epicardium of the left SVA, with severe narrowing of the LCX (Figure 3F). To rule out other etiologies, C-reactive protein, erythrocyte sedimentation rate, rheumatoid factor, antinuclear antibody, antidouble stranded DNA antibody, anti-neutrophil cytoplasmic antibody, lupus anticoagulant, T-spot, and syphilis serology tests were performed, but all were negative. Genetic testing revealed c.1781 C > G nonsense mutations in the *FLNA* gene. According to the American College of Medical Genetics and Genomics (ACMG) standard, *FLNA* c.1781 C > G was determined as a “likely pathogenic mutation” since it satisfied the criteria of both PVS1 (pathogenic very strong) and PM2 (pathogenic moderate).

**Figure 4:**
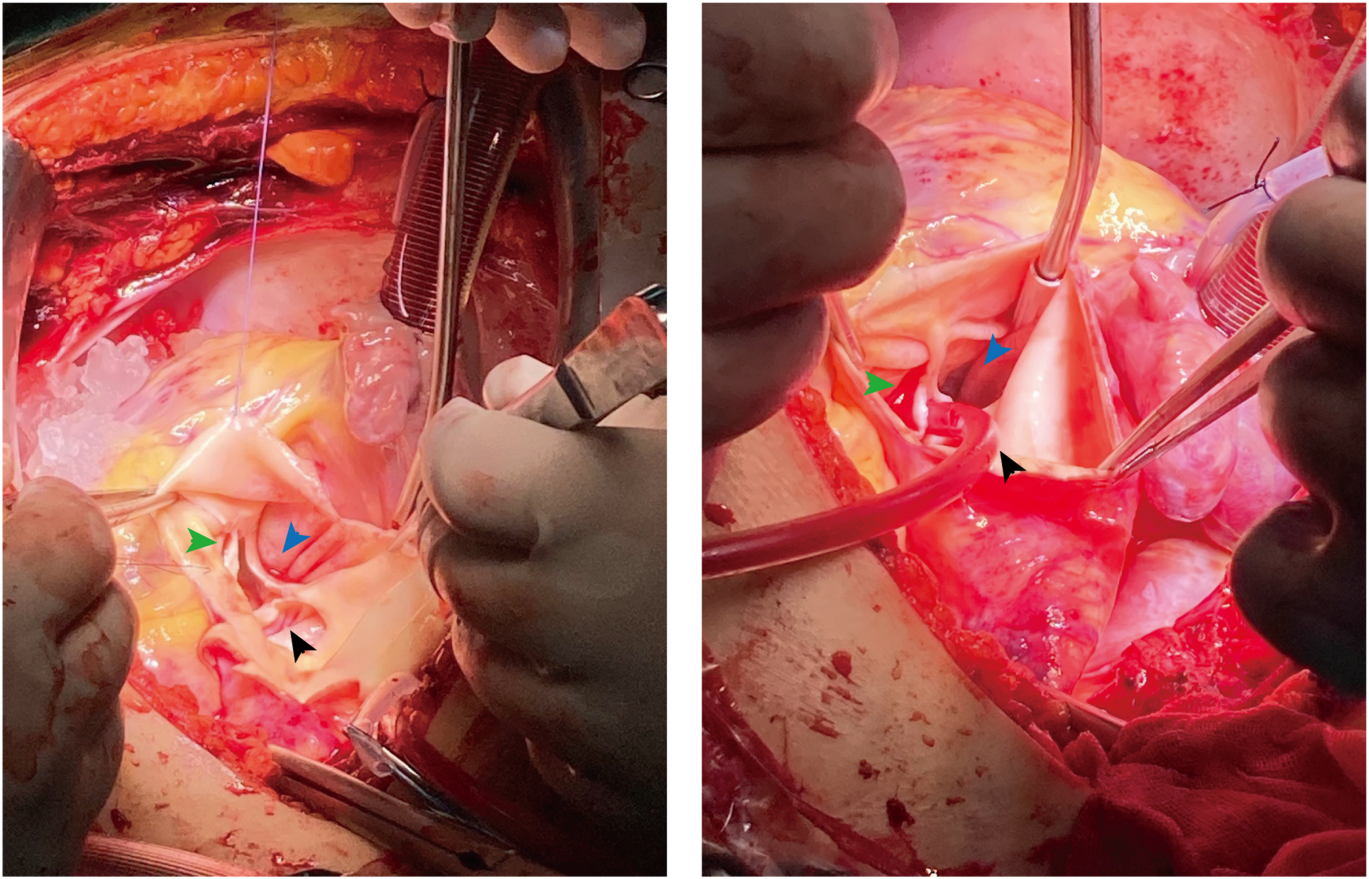
Intraoperative images confirmed the dual unruptured left (black arrow)/non-coronary (blue arrow) SVAs. The right coronary sinus (green arrow) was normal.

On admission, we administered nitroglycerin, an oxygen mask, and metachysis to alleviate her symptoms. Three days later, the patient underwent surgical intervention, including aneurysm patch closure, coronary artery reconstruction, and aortic valve and aortic root replacement. Operative findings confirmed a dual unruptured Valsalva aneurysm of the left coronary and non-coronary sinuses (Figure 4). The right coronary sinus was normal. The modified Bentall procedure was performed using a 26 mm biological composite graft and a mechanical aortic valve (Carbomedics Inc., Austin, TX, United States). The LM artery was then re-implanted into the newly formed left coronary sinus. Unfortunately, the patient experienced cardiac arrest during the operation, and, despite receiving an emergency thoracotomy, the heart was unable to recover after surgery and she died.

## Discussion

SVA is an uncommon congenital heart disease and its clinical symptoms depend on its origin and size, its influence on adjacent anatomical structures, and whether it ruptures. Regardless of etiology, the majority of SVA cases originate from the right coronary sinus (65%–86%), followed by the non-coronary sinus (10%–30%), while left coronary sinus aneurysms are rare (2%–5%) (6). However, cases of multiple SVAs are extremely rare (7). In our case of dual unruptured left/non-coronary SVAs, the dilated SVA compressed the LM, LCX, and pulmonary trunk, inducing postural angina pectoris and false “pulmonary hypertension”.

The etiology of SVA can be congenital or acquired, with the former being most common. Ventricular septal defects, bicuspid aortic valves, or aortic valve regurgitation often coexist with congenital SVA (8). In this case, echocardiography revealed a dilated aortic root and severe aortic regurgitation, while the aortic valve was tricuspid. Acquired SVA may be accompanied by manifestations of the primary diseases. In our case, none of the acquired pathologies, including chest trauma, infective disease, aortitis, or connective tissue disease, were found. However, a nonsense mutation of the *FLNA* gene, which encodes Filamin A and causes Melnick-Needles Syndrome and Cardiac Valvular Dysplasia, may account for the occurrence of SVA.

In previous studies, unruptured SVAs were generally asymptomatic and often presented as incidental findings during cardiac imaging, whereas those that ruptured into the cardiac chamber or pericardium induced acute heart failure or cardiac tamponade, respectively (9). Right SVAs protrude and rupture into the right ventricle, non-coronary SVAs tend to rupture into the right atrium, and left SVAs typically rupture into the pulmonary artery, left ventricle, or pericardium (10). Hemodynamic alterations can lead to relevant manifestations. Blood stagnation in the SVA leads to embolization, which may migrate the coronary or peripheral arteries (11). Several reports have described that left or right SVAs cause compression of the LAD, while non-coronary SVAs cause compression of the RCA, which results in angina pectoris (9, 12, 13). In our patient, the dilated left SVA compressed the LM and proximal LCX during systole, inducing acute interruption of coronary blood flow, which was relieved during diastole. Her symptoms were alleviated when she sat up due to the change in the direction of the effect of gravity on the left SVA. Dynamic electrocardiographic changes suggested an intrinsic mechanism: a mass effect of the left SVA on the LM and LCX since they run along the epicardium of the left SVA. In the supine position, the dilated left SVA compressed the epicardial coronary artery, impairing blood flow. However, the mass effect of the left SVA was relieved in the prone or upright and seated positions. CCTA clearly showed that the LM and proximal LCX narrowed and ran along the dilated left SVA. In the majority of relevant studies, coronary ischemia was caused by SVA compression; however, this study is the first to report that these symptoms are posture-dependent.

Patients with congenital heart diseases who develop pulmonary hypertension are always affected by left-to-right shunting such as atrial or ventricular septal defects. A ruptured right SVA fistulizing into the right atrium caused reversible flow-induced pulmonary hypertension (14). Mansour et al. (15) reported a very large right SVA compressing the right ventricular outflow tract that caused high right intraventricular pressure and tricuspid regurgitation in an elderly man. In our case, the dilated left and non-coronary SVAs did not rupture into the right heart system, and no crevasse was noted in the ventricular septum. Unusually, the unruptured left SVA grew sufficiently large to squeeze the pulmonary trunk, thus decreasing the pulmonary blood flow volume and pulmonary oxygenation. This is a case of false “pulmonary hypertension” in this patient as the right ventricular high pressure was caused by extrinsic compression of the pulmonary trunk. The oxygen-rich blood flow from the pulmonary capillaries to the peripheral arteries through the left heart was insufficient, which caused dyspnea and cyanosis.

The diagnosis and assessment of SVA relies on echocardiography, CCTA, and MRI (9). Three-dimensional images allow for a more accurate evaluation of aneurysms within the cardiac chambers and coronary arteries. Invasive angiography is risky in patients with SVA as their coronary arteries are difficult to engage, and their aortic roots are more prone to injury. Based on surgical series, case reports, and single-center data, early or emergent surgical repair is recommended in symptomatic unruptured and ruptured patients (16). As the unruptured SVAs caused postural angina pectoris and dyspnea in this case, we decided on surgical intervention immediately after systematic examination. Patch closure reduces the SVA volume, and aortic valve replacement or valvuloplasty is necessary in cases of aortic valve regurgitation. Other incisions are performed when the SVA ruptures into the cardiac chambers. Coronary artery bypass grafting should be performed in cases of an SVA-compressed epicardial coronary artery. Reparative surgery is associated with a low perioperative mortality rate, a low risk of recurrence, and long-term survival (5). Percutaneous aneurysm closure devices have been shown to be an alternative treatment with promising results (17).

Unfortunately, our patient experienced cardiac arrest during the operation. Several issues might have caused the death: (1) the dilated left SVA might have severely compressed the coronary arteries during anesthesia and thoracotomy, which might have induced acute myocardial infarction. Inadequate cardiac output might have led to circulatory collapse. (2) The pulmonary trunk was completely obstructed by the SVA. There was no oxygen-rich blood coming from the pulmonary circulation to the heart, which might have induced cardiac arrest and oxygen depletion. (3) Anatomically, the atrioventricular node is close to the non- and right-coronary sinuses. Electrocardiography revealed 1st atrioventricular block in this patient on admission. We presume that the non-coronary SVA might have compressed the atrioventricular node and might have induced complete atrioventricular block during the operation.

In conclusion, SVA is a rare cardiac abnormality with several clinical manifestations. This case suggests that, in patients with unexplained postural angina pectoris or pulmonary hypertension, the possibility of SVA should be considered. Echocardiography should be immediately performed in suspected cases. A prompt diagnosis can be established based on CCTA findings. Once identified, immediate surgical intervention as soon as possible is recommended.

## Data Availability

The original contributions presented in the study are included in the article/**Supplementary material**, further inquiries can be directed to the corresponding author.
